# Researches into the Physical History of Mankind

**Published:** 1838-04

**Authors:** 


Art. I.—*Researches into the Physical History of Mankind.* By James Cowles Prichard, m.d. f.r.s. &c. &c. *Third Edition.* Vol. II.; *containing Researches into the Physical Ethnography of the African Races.—London,* 1837. 8vo. pp. 373.

The first volume of this work has been already briefly noticed by us: the second, now before us, contains a continuation of the enquiry into the relation of the human races to each other, as exemplified in those of all parts of the vast continent of Africa. In the previous portion of the work Dr. Prichard endeavoured to shew "that no remarkable instance of variety in organization exists among the human races, to which a parallel may not be found in many of the inferior tribes;" and also, "that all human races coincide in regard to many particulars, in which tribes of animals, when specifically distinct, are always found to differ." He proceeds in this volume to investigate the nature of the organic diversities in mankind in a different way, and to enquire how far the characters of particular tribes enquired into have been permanent, or in what respects subject to variations. The question here to be decided is, whether the distinguishing characters of the human races have been constant and undeviating within the period of time to which historical testimony extends. That they have been thus constant and undeviating is, Dr. Prichard observes, a very prevalent opinion, and is strongly opposed to the admission of the unity of the species of mankind; and, to decide this important point, he enters on an investigation of the physical history of particular races of men or families of nations.

Dr. Prichard considers it altogether hypothetical to divide mankind, as has been so often done, into a few classes or groups resembling each other in physical character, and to assume that such groups constitute races or lineages, of which the members are more nearly allied than to tribes of different physical peculiarities. Avoiding all attempts to distribute mankind into departments on principles purely conjectural, he sets forth his intention to be to "proceed in a geographical arrangement, to examine the phenomena which present themselves in the population of different regions of the world." In this manner he is of opinion that an opportunity is also afforded of more correctly marking the effects of physical agencies in the development of varieties of breeds, or in the origination of new or diversified races. But many circumstances, he remarks, must be taken into such an investigation beyond the relative distance of different climates from the poles or the equator. There are conditions to be taken into the consideration which often differ much in the same latitudes. Dr. Prichard, therefore, endeavours to ascertain "what are the most remarkable features in the physical geography of each region, and what relations the origin and development of varieties in families or tribes may bear to all these local conditions."

The reader who feels desirous of imformation on this most interesting subject cannot peruse with too much attention this most important and difficult part of Dr. Prichard's subject, relating to the races which constitute the population of Africa. He will there find many remarkable and some unexpected particulars, calculated, we think, not only to throw much light upon the enquiry which they principally illustrate, but to enlarge our conceptions of the capacities and destinies of some portions of mankind, of which the inferiority has been asserted on insufficient foundation, and acted upon without kindness or humanity. He will also, we think, see abundant proofs that the characteristic qualities of human races are *not* permanent and undeviating. Of the various facts accumulated in this volume, and by which the very learned and able author establishes these points, no analysis or abridgment could give the reader an adequate idea. The whole work must be carefully gone through, in order to appreciate the perfection of the powerful chain of reasoning which binds observations so extended and so various into satisfactory proofs of the doctrines propounded.

